# Collision tumour involving a rectal gastrointestinal stromal tumour with invasion of the prostate and a prostatic adenocarcinoma

**DOI:** 10.1186/1746-1596-7-150

**Published:** 2012-10-30

**Authors:** Laura Macías-García, Haydee De la Hoz-Herazo, Antonio Robles-Frías, María J Pareja-Megía, Juan López-Garrido, José I López

**Affiliations:** 1 Unidad de gestión clínica de Oncohematología y Anatomía Patológica, Hospital Universitario de Valme, Carretera de Cádiz s/n, 41014, Sevilla, Spain; 2 Department of Anatomic Pathology, Hospital Universitario Cruces, Instituto BioCruces, University of the Basque Country, Barakaldo, Bizkaia, Spain

**Keywords:** Gastrointestinal stromal tumour, Mixed tumours, Prostatic adenocarcinoma, Imatinib

## Abstract

**Background:**

Gastrointestinal stromal tumours (GISTs) are the most common primary mesenchymal neoplasia in the gastrointestinal tract, although they represent only a small fraction of total gastrointestinal malignancies in adults (<2%). GISTs can be located at any level of the gastrointestinal tract; the stomach is the most common location (60-70%), in contrast to the rectum, which is most rare (4%). When a GIST invades into the adjacent prostate tissue, it can simulate prostate cancer. In this study, we report on a case comprising the unexpected collision between a rectal GIST tumour and a prostatic adenocarcinoma.

**Findings:**

We describe the complexity of the clinical, endoscopic and radiological diagnosis, of the differential diagnosis based on tumour biopsy, and of the role of neoadjuvant therapy using imatinib prior to surgical treatment.

**Conclusions:**

Although isolated cases of coexisting GISTs and prostatic adenocarcinomas have previously been described, this is the first reported case in the medical literature of a collision tumour involving a rectal GIST and prostatic adenocarcinoma components.

**Virtual slides:**

The virtual slide(s) for this article can be found here: http://www.diagnosticpathology.diagnomx.eu/vs/1238437468776331.

## Background

The term gastrointestinal stromal tumor (GIST) was first introduced in 1983 by Mazur and Clark as a neutral term between smooth muscle tumours and schwannomas. GIST is currently used to refer to a heterogeneous group of mesenchymal tumour lesions, acquired or congenital, with common morphological profiles that usually settle within the digestive tract.

Although GISTs are the most common primary mesenchymal tumours of the gastrointestinal tract (70%), they represent only a small fraction of all gastrointestinal malignancies in adults (<2%).

GISTs usually affect adults between 6th and 8th decades of life without specific gender prevalence (M:F, 1.1:1) [1]. Its annual incidence rates range from 6.8 and 14.5 cases per million in the U.S. States and in Sweden, respectively [2]. GISTs include benign and malignant neoplasia that immunohistochemically stain positively for KIT (CD117) and that phenotypically differentiate into cells of Cajal. The histological (e.g., cellularity spindle and/or epithelioid) and immunohistochemical (e.g., CD117 and CD34 immunostaining) profiles allow for simple morphological diagnosis.

In 1998, Hirota et al. showed that the majority of GISTs exhibit activating mutations in the KIT protooncogene. Subsequently, in 2003, a new mutation in PDGFRA and the sensitivity of GISTs to tyrosine kinase inhibitors such as imatinib were demonstrated. These findings represent an important advance in the clinical management and our understanding of the biology of this tumour [2]. Although the mutations in KIT and PDGFRA represent the basis of GIST oncogenesis, 5-10% of the GIST cases are negative for mutations in KIT [3].

GISTs can be located at any level of the gastrointestinal tract. The stomach is the most common location (60-70%), whereas rectal GIST represents only 4% of all GISTs [1]. When these tumours invade into the prostate, they can clinically simulate a prostatic adenocarcinoma [4]. The direct invasion of the prostate by a rectal GIST is rare [4-7] and the case presented in our study involves an added diagnostic complexity: a collision tumour involving both histological types of neoplasia. The combination of a rectal GIST that invades into the prostate and a primary prostatic adenocarcinoma in a collision tumour has never before been described in the medical literature.

## Materials and methods

### Case presentation

The patient examined in this study was a 52-year-old male with no relevant personal or family history of disease, who presented with rectal tenesmus, rectal bleeding, and obstructive urinary symptoms. A digital rectal exam revealed a fixed submucosal mass in the anterior rectal wall, attached to the prostate and located 2–3 cm from the anal margin. The prostate specific antigen (PSA) value was 3.16 mg/ml, with a pathologic PSA ratio of 0.0937. Rectoscopic and echo-endoscopic evaluations revealed a rectal neoplasia with prostatic infiltration. A computed tomography (CT) scan performed at 5 months prior to the surgical intervention revealed a relatively well-defined heterogeneous mass of 60 mm × 36 mm × 45 mm affecting the anterior wall of the rectum in contact with the prostate, with effacement of the fat separating the two organs.

Magnetic resonance imaging (MRI) performed at the same time revealed a large (7–8 cm) tumour in the distal rectum, affecting the adjacent prostate and causing stenosis of the rectal lumen. The CT scan and the MRI results both revealed an absence of metastasis in the local and distant lymph nodes.

A biopsy of the tumour revealed a profile that was consistent with a mesenchymal neoplasia of uncertain malignancy that was positive for CD117 (cytoplasmic and membranous staining), the expression of which is characteristic of GISTs.

The treatment protocol consisted of neoadjuvant treatment with imatinib abdominoperineal rectal amputation with a radical cystectomy and prostatectomy. A preoperative CT scan demonstrated an apparent reduction in the tumour size, measuring 50 mm × 30 mm, where the separation between the rectum and prostate was difficult to delineate.

### Pathologic evaluation

**Figure 1:**
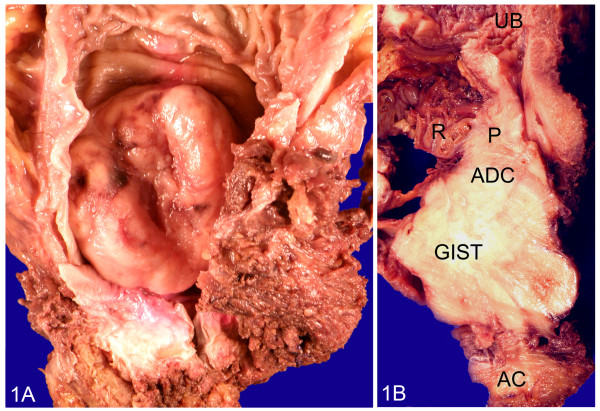
**A: Rectal GIST: Excrescentand ulcerated rectal masswith stenosis of therectal lumen.B**: Rectal GIST with prostatic infiltration: rectal tumour with direct invasion of both adjacent lobules of the prostate. UB: urinary bladder; ADC: prostatic adenocarcinoma; AC: Anal canal; R: rectum; P: prostate.

**Figure 2:**
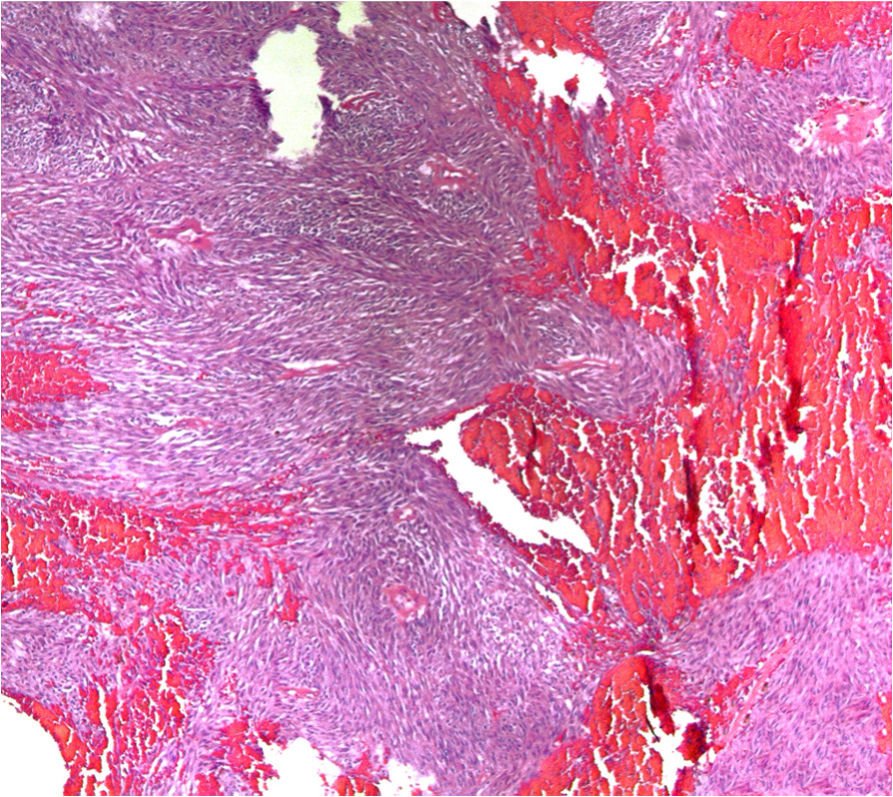
**Rectal GIST: Spindle celltumour with haemorrhagic foci.HE (100x).**

**Figure 3:**
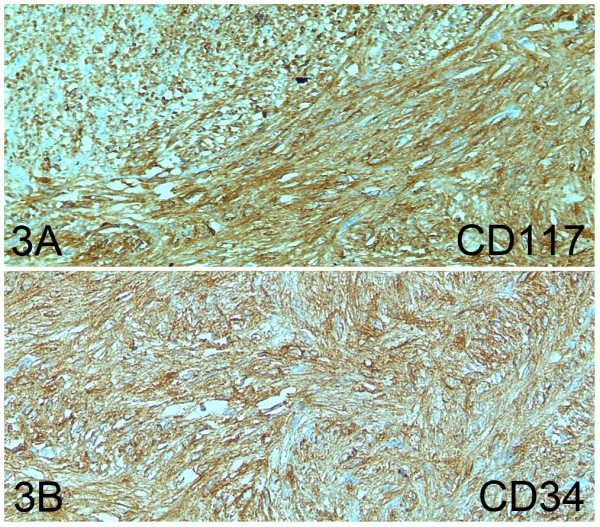
**A, B: Rectal GIST:Positive immunostaining with CD117(cytoplasmic and membranous) andCD34.**

Grossly, the surgical specimen was an excrescent mass located within the anterior rectal wall that exhibited a 7-cm maximum diameter and focally ulcerated mucosa (Figure 1: A). An incision revealed a fleshy appearance with haemorrhagic foci and infiltration into of over half of both lobes of the prostate (Figure 1: B). Histologically, we observed spindle tumour cells with moderate atypia that were arranged in a fascicular pattern, as well as extensive haemorrhagic areas without relevant necrotic foci (Figure 2). The mitotic index was 18/50 high-power field (HPF). We observed no other regressive changes attributable to treatment with imatinib, except for the haemorrhagic foci. The immunohistochemical profile of the spindle cell tumour was as follows: cytoplasmic and membrane CD117+, CD34+ (Figure 3: A, B), vimentin+, actin HHF-35-, actin SMA-, desmin-, and S-100-, and a Ki-67 proliferative index of 15%.

**Figure 4:**
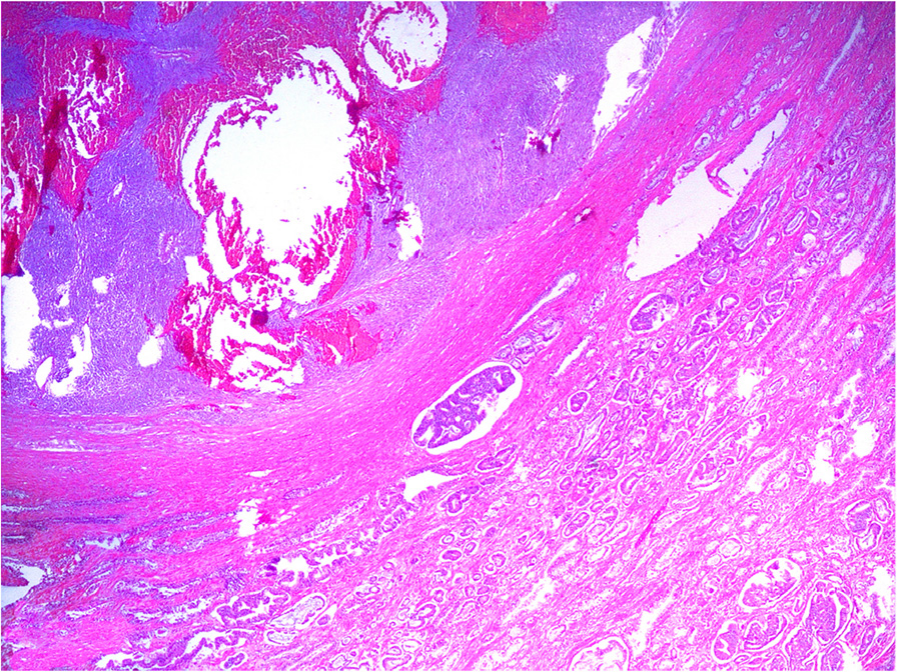
**Collision tumour rectal GIST/prostaticadenocarcinoma. HE (50x).**

The prostate tissue adjacent to the infiltrative front of the spindle cell tumour exhibited diffuse proliferation of microglandules and well-defined cribriform structures with focal intraluminal mucin secretion and atypical cells with prominent nucleoli (Figure 4).

Histopathological diagnosis identified the collision tumour involving a high-risk GIST of the spindle cell variant that originated in the anterior rectal wall and that exhibited perirectal extension and wide infiltration of the adjacent peripheral prostate lobules, as well as a prostatic acinar adenocarcinoma with a combined Gleason score of 6 (3+3) and confined involvement to the left and right prostate lobules (pT2c). The seminal vesicles and urinary bladder exhibited no evidence of neoplasia.

Currently, after 8 months of postoperative follow-up and neoadjuvant treatment with imatinib, the patient is asymptomatic and cancer-free.

## Discussion

GIST is the most common type of non-epithelial neoplasia diagnosed in the gastrointestinal tract (0.1-3% of all gastrointestinal tumours) with an incidence rate of 10–20 cases per million population [8]. However, these tumours rarely occur in the rectum; the reported incidence rate is approximately 1 case per 1000 GISTs [7]. The presentation of these tumours can range from asymptomatic nodules smaller than 1 cm that are incidentally diagnosed in the surgical specimens of rectal resections to masses larger than 10 cm (“giant GISTs”) that result in altered intestinal function, rectal bleeding in the case of ulceration, abdominal pain, and urinary symptoms attributed to bladder compression [9]. When located in the anterior wall of the rectum in male patients, such tumours might cause difficulties in urination along with pelvic and perineal pain, producing symptoms similar to prostatism. In rare cases, the tumour can extend into the prostate gland, clinically and radiologically simulating prostate cancer [4]. Histologically, rectal GISTs tend to contain large spindle tumour cells that are often arranged in palisades.

The immunohistochemical profile of the tumour was similar to gastric GISTs. As many as 90-100% of tumour cells exhibited strong immunopositivity for CD117, DOG-1, and CD34 and were generally immunonegative for SMA and S-100. These tumours behave in an aggressive fashion, exhibiting a high rate of recurrence, pelvic extension, and hepatic and bone metastases [1].

The differential diagnosis of rectal GIST with prostatic involvement in the biopsy specimens is primarily based on immunohistochemical evaluation and includes different stromal neoplasias with spindle cell patterns that can affect the rectum and prostate. These tumours include primarily smooth muscle tumours (leiomyomas, leiomyosarcomas), schwannomas, fibromatosis, a solitary fibrous tumour and a malignant melanoma. The differential histological and immunological characteristics of the tumours localised in this region are summarised in (Table 1).

**Table 1:** **Histological and immunohistochemical differentialdiagnosis of rectal GISTinvolving prostate**

**Diagnosis**	**GIST (rectum/ prostate)**	**Leiomyoma (rectum/prostate)**	**Leiomyosarcoma (rectum/prostate)**	**Schwannoma (rectum/prostate)**	**Fibromatosis (mesenteric)**	**Solitary fibrous tumour (prostate)**	**Malignant melanoma (rectum/ prostate)**
**Growth Pattern**	Fascicles, storiform or palisading	Fascicles	Fascicles	Antoni A/ Antoni B	Parallel fascicles, collagenous background	“Patternless pattern”, collagenous background, staghorn vessels	Diffuse / nested
**Cell Type**	Spindle cell, high cellularity, no perinuclear vacuolization	Spindle cell, “cigar-shaped” nuclei, perinuclear vacuolization, low cellularity	Spindle cells, “cigar-shaped nuclei, atypia and intense nuclear pleomorphism, vacuolization, high cellularity	Spindle cells, wavy nuclei, nuclear atypia may be present	Bland spindle or strellate-cells, low to moderate cellularity	Spindle cells, low or high cellularity, pleomorphism (variable)	Epithelioid (rarely spindle cell)
**Mitosis**	Few to many	Few	Frequent	Rare	Rare	0-15 / 10 HPF	Frequent
**Necrosis/ Hemorrhage**	May be present	Absent	Present	Sometimes present	Absent	Variable	Usually present
**CD 117**	Consistenly positive (cytoplasmic and membranous)	Negative	Negative	Negative	Positive sometimes (cytoplasmic)	Negative	Rarely positive
**CD34**	Consistenly positive	Rarely positive	Negative	Sometimes focal	Very rare	Positive	Negative
**Actina (SMA)**	Sometimes positive	Positive	Positive	Negative	Positive	Rarely	Negative
**Desmina**	Rarely	Positive	Positive	Negative	Very rare	Negative	Negative
**S-100**	Rarely	Very rare	Very rare	Strongly positive	Negative	Negative	Positive
**HMB-45**	Negative	Negative	Negative	Negative	Negative	Negative	Positive
**Beta-Catenin**	Negative	Negative	Negative	Negative	Positive	Negative	Negative

To confirm the diagnosis of a GIST, a KIT or PDGFRA mutational analysis can be performed, although this was not a possible option in our patient.

GIST is known to coexist with certain neoplasias, including pulmonary chondromas and paragangliomas (Carney’s triad). For the diagnostic of CT (Carney´s triad) are necessary two of the three components. The association of GIST and pulmonary chondroma is the most common combination [10]. Sporadic GISTs of the adults are completely different from CT-GISTs and pediatric GISTs. Regional lymph nodes are frequently metastasize by both GISTs (29%), while their occurrence in common GISTs seen in adults is rare (2%) [10]. Furthermore, multiple types of GISTs have been associated with type I neurofibromatosis.

However, these cases have been described as synchronous or metachronous association between sporadic GISTs and other neoplasia [11,12], with a mean secondary tumour incidence rate of 9.3%, according to the data from 15 different previous studies including 4,777 GIST patients [13], and up to 13.8% according to data from a recent study [14]. The majority of secondary tumours described were gastrointestinal adenocarcinomas, although a large study also reported a relative 9% incidence of prostatic adenocarcinoma [11].

Radical cystoprostatectomy performed due to bladder carcinoma showed prostatic acinar adenocarcinoma in the 49.6% of the cases [15], where 81.3% of these cases represented clinically insignificant prostatic adenocarcinomas. However, in this study, the prostatic tumour was extensive and multifocal, with peripheral affectation of the prostate lobules and exhibiting a relevant Gleason score (3 + 3). The tumour exhibited no apparent contacts with the surgical margins and no extraprostatic extensions. However, in the regions of collision with the rectal GIST, it was not possible to determine the precise extent of the tumour.

Rectal GISTs are rare tumours, whereas prostatic adenocarcinoma is the most prevalent type of non-cutaneous neoplasia in elderly men. On this basis, rectal and prostate tumours are generally initially considered as prostatic adenocarcinomas or prostatic infiltrations of rectal adenocarcinomas. Only 6 cases have been described of prostatic infiltration by rectal GISTs [4-7], all of which were initially diagnosed as primary prostatic leiomyosarcomas by evaluation of transrectal biopsy. Surprisingly, GISTs have also been described as a type of primary prostatic tumour [16]. In all cases, the final diagnosis is established by analysing the surgical specimen. In our study, the collision of two histologically distinct tumour types increased the complexity of the clinical, radiological, and histological diagnosis. This is the first case describing a collision tumour involving rectal GIST with direct prostatic invasion and a prostatic adenocarcinoma. This pathological situation greatly confounded the clinical diagnosis, particularly given the presence of a rectal mass, rectal and prostatic symptoms, and pathological levels of PSA.

Until recently, the treatment of giant rectal GISTs has been limited to surgical resection, except for cases of inoperable tumours or metastases that contraindicated the surgical treatment (indicating that the tumour was incurable under those specific circumstances). The use of imatinib as a neoadjuvant treatment offers the possibility of a partial tumour regression and stabilisation of the disease [9]. In general, the best therapeutic results have been obtained in tumours with KIT mutations in exons 11 (85%) and 9 (45%) [3], although a global response rate of only 40% in inoperable tumours indicates that resection is a non-curative procedure in many cases [17].

In our study, neoadjuvant therapy with imatinib did not substantially reduce the tumour size, and histologically, we only observed extensive haemorrhagic foci without a decrease in the number of tumour cells or the presence of myxohyaline stroma, necrosis, or cystic degeneration, as previously described in cases that exhibited an initial response to treatment [3]. However, both tumours were completely resected, and after 8 months of postoperative follow-up and adjuvant therapy with imatinib, the patient remains asymptomatic and cancer-free.

## Consent

The authors obtained the rights for publication of this report and any accompanying images.

## Abbreviations

GIST: Gastrointestinal stromal tumours; PSA: Prostate specific antigen; CT: Computed Tomography; MRI: Including Magnetic Resonance; HPF: High-Power Field.

## Competing interests

The authors declare that they have no competing interests.

## Authors’ contributions

All authors read and approved the final manuscript.
